# Proprioceptive Neuromuscular Facilitation-Based Physical Therapy on the Improvement of Balance and Gait in Patients with Chronic Stroke: A Systematic Review and Meta-Analysis

**DOI:** 10.3390/life12060882

**Published:** 2022-06-13

**Authors:** Phan The Nguyen, Li-Wei Chou, Yueh-Ling Hsieh

**Affiliations:** 1 Department of Physical Therapy, Graduate Institute of Rehabilitation Science, China Medical University, Taichung 406040, Taiwan; phanthenguyen23@gmail.com (P.T.N.); chouliwe@mail.cmu.edu.tw (L.-W.C.); 2 Department of Physical Therapy, Faculty of Nursing and Medical Technology, University of Medicine and Pharmacy, Ho Chi Minh City 8428, Vietnam; 3 Department of Physical Medicine and Rehabilitation, China Medical University Hospital, Taichung 406040, Taiwan; 4 Department of Rehabilitation, Asia University Hospital, Taichung 413505, Taiwan

**Keywords:** stroke, postural balance, gait, proprioceptive neuromuscular facilitation, stroke rehabilitation

## Abstract

The present study aims to determine the potential benefits of PNF on balance and gait function in patients with chronic stroke by using a systematic review and meta-analysis. Systematic review in the following databases: MEDLINE/PubMed, Physiotherapy Evidence Database (PEDro), Cochrane Library and Google Scholar. Studies up to September 2020 are included. A systematic database search was conducted for randomized control trials (RCTs) that investigated the effects of PNF intervention in patients with chronic stroke using balance and gait parameters as outcome measures. The primary outcomes of interest were Berg Balance Scale (BBS), Functional Reach Test (FRT), Timed Up and Go Test (TUG) and 10-Meter Walking Test (10MWT). Nineteen studies with 532 participants were included, of which twelve studies with 327 participants were included for meta-analysis. When the data were pooled, PNF made statistically significant improvements in balance with BBS, FRT and TUG (*p <* 0.05) or gait velocity with 10MWT (*p <* 0.001) when compared to the control. This review indicates that PNF is a potential treatment strategy in chronic stroke rehabilitation on balance and gait speed. Further high-quality research is required for concluding a consensus of intervention and research on PNF.

## 1. Introduction

Stroke survivors in the chronic stage, namely, more than six months after stroke onset, often have long-term residual and disabling deficits, especially on the impairment of motor tasks [1,2,3]. Moreover, muscle coordination is often decreased after a stroke, leading to deficits for both gait and balance control [4]. Even for stroke patients with independent motor functions, balance impairments and gait abnormalities are two of the most common manifestations in the chronic stage [5]. These deficits severely impede the individuals’ abilities to participate in activities of daily living and negatively impact their quality of life. In addition, they indicate an increased risk for falls and a greater likelihood of hospital or nursing-home admissions [5].

Several motor functions can often be improved with time and through various rehabilitation strategies, including modality, manual and movement therapies. However, the asymmetric postural behavior of stroke survivors during standing and walking is often reinforced, maintained or only transitorily decreased in the chronic recovery stage [4,6]. Asymmetric posture can also be due to impaired sensory inputs, including decreased perception of midline. Increased use of the unaffected side can be a result of this as well [7]. In view of this, chronic stroke rehabilitation, including muscle re-education in both affected and unaffected sides, should first emphasize the correction of the postural asymmetric pattern by enhancing the balance control of particular motor tasks beneficial to gait.

There are many available modality and movement therapies of post-stroke rehabilitation that have positive effects on motor and gait functions in patients after stroke, e.g., cycling, treadmill walking and functional electrical stimulation [8,9]. However, they may be expensive and provide a limited kind of movement. Proprioceptive neuromuscular facilitation (PNF) is a therapeutic approach that uses cutaneous, proprioceptive and auditory input to produce functional improvement in motor output and can play a vital role in the rehabilitation of many injuries. It is a specific manual technique controlled by physical therapists to help improve a patient′s functional status by incorporating multiple planes of movements, making the task more functional and effective in achieving patient goals. As it exhibits effects on the improvement of pain, range of motion, muscle strength and endurance, coordination and facilitation of proximal stability and functional progression, it has been widely used for early rehabilitation of the acute or subacute phases for neuromuscular re-education to improve motor functions of patients with stroke [10,11]. This method stimulates proprioceptive organs in muscles and tendons to improve muscular functions, promotes the exploration of postural reflexes and prioritizes muscle contraction for increasing strength, flexibility, balance and coordination [12,13,14]. Preliminary case reports revealed that a PNF-based program has the potential to generate positive outcomes on motor function in older adults with chronic stroke [12,15,16]. Two systematic reviews with small samples (five and twelve studies, respectively) reported that PNF is an effective treatment for improving gait-related outcome measures in patients with stroke [11,17]. However, despite an increase in the published literature on the effects of PNF, there is still limited evidence from the meta-analysis of randomized-controlled trials (RCTs) to quantify the efficacy of PNF-based approaches on the improvement of motor outcomes, especially for balance and gait in patients with chronic stroke.

A concise and up-to-date overview of the effectiveness of PNF-based training on balance and gait in patients with chronic stroke is currently lacking. This study is a systematic and meta-analytical review of the available RCTs to examine the effects of PNF on improvements of balance and gait functions in patients with chronic stroke only, excluding those in acute and subacute phases after stroke.

## 2. Materials and Methods

A systematic review was conducted in accordance with the PRISMA guidelines [18] and the review protocol was registered with OSF (URL: https://osf.io/p26rj (accessed on 27 October 2020)).

### 2.1. Search Strategies

A systematic search of the related literature published between 1960/01 and 2021/07 was performed in MEDLINE/PubMed, Physiotherapy Evidence Database (PEDro), Cochrane Library, Google Scholar, CINHAL, Web of Science and China Knowledge Resource Integrated (CNKI). Database-specific filters were used, as necessary, to complete searches in all the specified databases. Search filters were utilized so that only human studies and RCTs were included. Hand searches were completed using the reference lists of related articles. No language or date restrictions were imposed on the search. The search strategies and keywords are illustrated in Table 1.

### 2.2. Inclusion/Exclusion Criteria for Studies

The inclusion criteria were as follows: studies of adult patients (>18 years old) diagnosed with chronic stroke (more than 6 months after stroke onset), studies using PNF alone or in combination with other therapies as intervention in experimental groups and non-PNF-based intervention in control groups, studies that measured balance- and gait-related changes, and studies that used a RCT design. The exclusion criteria were graduation theses, books, conference proceedings, including posters and platforms, single case studies, quasi-randomized clinical trials and qualitative studies. This systematic review protocol followed the recommendations of the Preferred Reporting Items for Systematic Review and Meta-Analysis (PRISMA) Protocols [18].

### 2.3. Screening Process

The initial identification and selection of studies in the electronic search was conducted by two reviewers, who independently evaluated the titles and abstracts of all primary studies. Then, the reviewers chose texts that were considered as eligible references and evaluated whether they met the pre-established inclusion criteria. In case of a disagreement between the two reviewers, the third reviewer would make the final decision.

### 2.4. Methodological Quality and Risk of Bias Assessment

The methodological quality of the studies was evaluated using the 11-item PEDro Scale. Studies with PEDro scale scores of 9–10 were considered to be of excellent quality, those with scores of 6–8 and 4–5 were of good and fair quality, respectively, and those with scores below 4 were of poor quality [19]. The PEDro score demonstrated “fair” to “excellent” inter-rater reliability (Intraclass Correlation Coefficient 0.53–0.91) for RCT of physiotherapy interventions [20]. For studies not published on the PEDro database, but that met the inclusion criteria, two reviewers would independently assess the quality of these studies using the PEDro scale. The Cochrane Handbook for Systematic Reviews of Interventions (Cochrane Collaboration) was used to evaluate the risk of bias in the included trials (reported as low, high or unclear risks). The following criteria were used to assess the risk of bias: the generation of randomization sequence, allocation concealment, blinding, completeness of the data and reporting of outcomes. In case of a disagreement in the quality assessment between the independent reviewers, a consensus was reached by a discussion or consultation with a third reviewer of the research team.

### 2.5. Data Extraction and Analysis

Study characteristics, participant characteristics, interventions, duration of trial period, period of outcome assessment and main results were extracted from the selected studies. A meta-analysis of the study was performed using RevMan software, version 5.3 (The Nordic Cochrane Centre, The Cochrane Collaboration, Copenhagen, Denmark). Outcomes and results were described. Meta-analysis was conducted on outcome measures used in at least three RCT studies to assess the balance and gait. The pooled effect estimates were computed with a random-effect model using the means, standard deviations (SDs) of the post-intervention and number of participants [21]. Heterogeneity was assessed using I^2^ statistics. Sensitivity analysis was conducted to assess the influence of each study on the combined results by excluding individual trials one at a time. Pooled-effect estimates were obtained by comparing the change in the least square mean from baseline to endpoint for each group, and were expressed as the mean difference (MD) with a 95% confidence interval (CI) between groups. A *p*-value < 0.05 indicated a significant statistical difference.

## 3. Results

### 3.1. Study Selection

**Figure 1:**
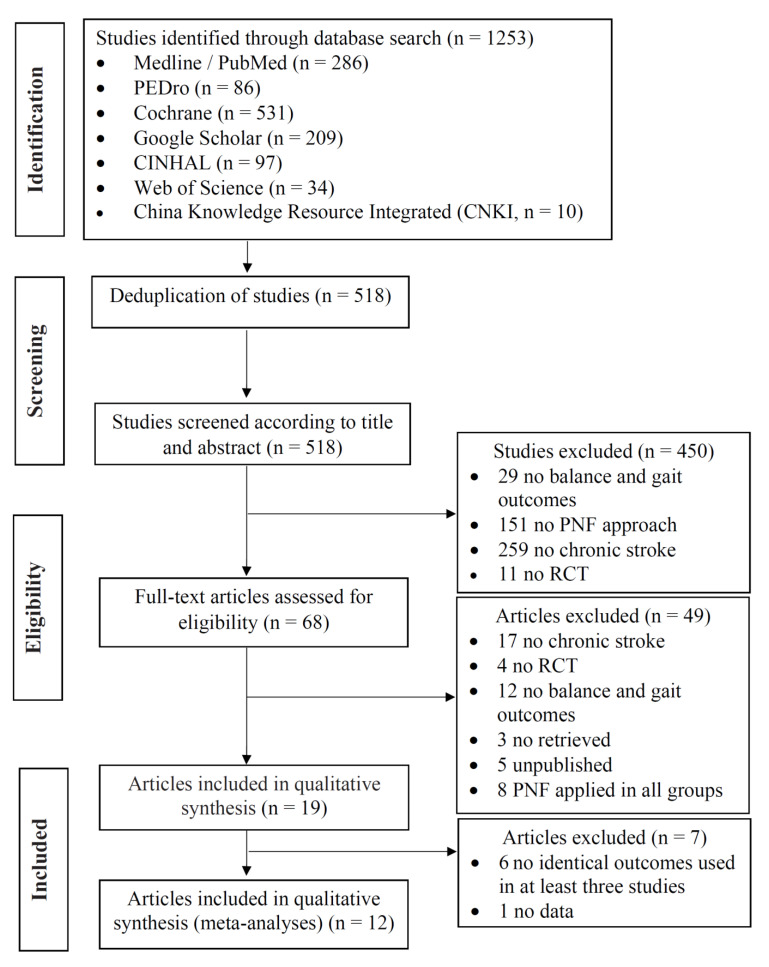
Flowchart for study selection for systematic review and meta-analysis.

A total of 1253 potentially relevant studies were identified from the databases f the initial search. Following deduplication, 518 articles underwent title and abstract screening. In the end, 68 articles were included for full-text review, which further excluded 49 articles, with the remaining 19 studies for qualitative synthesis [22,23,24,25,26,27,28,29,30,31,32,33,34,35,36,37,38,39,40]. Screening the evaluation instruments used showed that the Berg balance scale (BBS), functional reach test (FRT), timed up-and-go test (TUG) and 10-m walking test (10MWT) were used in at least three separate studies, and their data in a total of 12 articles were combined for quantitative review by meta-analysis. Figure 1 depicts the PRISMA flowchart.

### 3.2. Quality Appraisal and Risk of Bias

**Figure 2:**
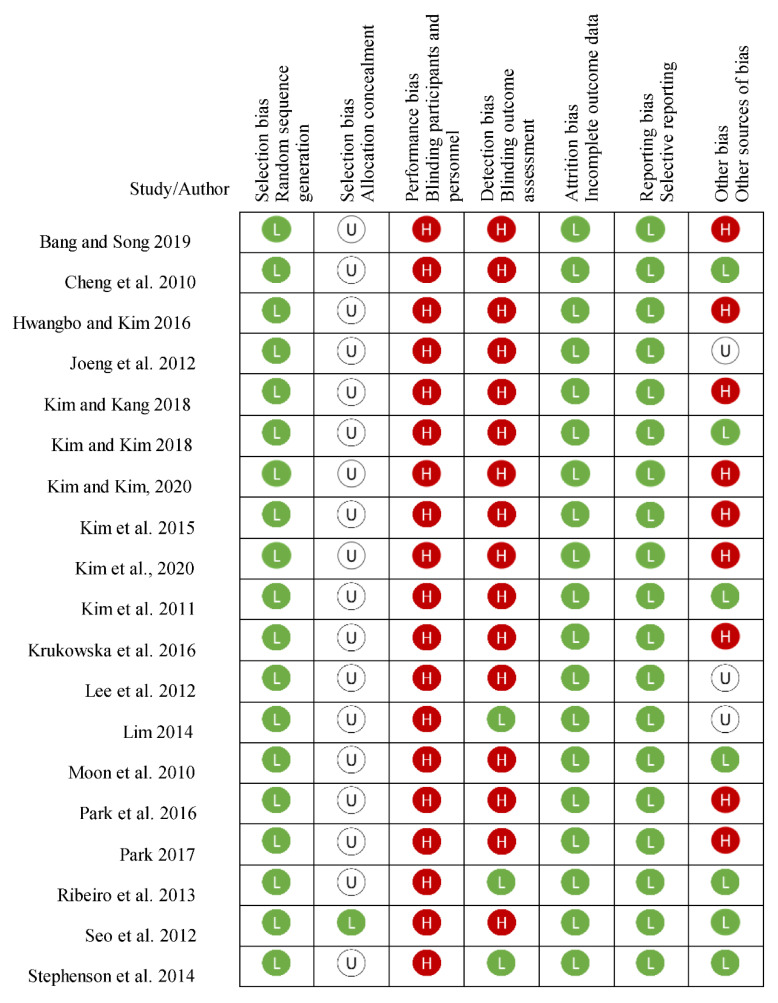
The risk of bias assessment summary using the Cochrane Risk of Bias Assessment tool. The L sign indicates a low risk of bias, H indicates a high risk of bias and U sign indicates an unclear risk of bias [22,23,24,25,26,27,28,29,30,31,32,33,34,35,36,37,38,39,40].

Table 2 shows the quality assessment of included studies according to the 19 items of the PEDro scale. The PEDro scores of these 19 studies ranged from 5 to 7, with an average of 5.43 ± 0.62. As can be observed, 6 studies with scores of 6 or 7 are of good methodological quality with low risk of bias [27,34,35,38,39,40]. PEDro scale items, namely, “Randomization”, “Comparison at the baseline”, “Adequate follow-up”, “Comparisons between groups” and “Measures of precision and variability”, were involved in the research designs of all included studies. The most common reasons for lower scores with risk of bias were the lack of “Allocation concealment” in 18 studies, “Blind participants” in the 19 included studies, “Blind therapists” in 18 studies, “Blind evaluators” in 16 studies and “Intention-to-treat analysis” in 17 studies (Table 2). The risk of bias in all studies did not achieve the low bias level and there were some concerns for all trials from the results evaluated using the Cochrane Risk of Bias Assessment tool (Figure 2).

### 3.3. Participant Characteristics

The included studies had a total of 532 participants, with the sample size of each RCT ranging from 10 to 72 participants. The mean age of participants of both genders ranged from 34.3 to 83.6 years in the experimental groups and from 36.2 to 80.1 years in the control groups. Types of stroke included hemorrhagic and ischemic strokes. Time since stroke onset ranged from 7.6 to 69.4 months in the experimental groups and from 6.3 to 81.49 months in the control groups.

### 3.4. Participant Inclusion Criteria

All studies selected patients with chronic stroke who had been diagnosed with stroke at least six months earlier as a primary inclusion criteria. Of the nineteen studies analyzed, fifteen studies used stroke at the chronic stage for those who could understand and follow the researcher′s instructions according to Mini-Mental State Examination scores (MMSE > 20 points) [22,23,24,25,26,27,28,30,31,34,35,36,39]. Two studies used the Brunnstrom scale [35,37] and three studies used the Modified Ashworth Scale (MAS) [25,31,38] to describe the spastic and involuntary muscle movement of lower limbs as inclusion criteria of a participant’s motor severity. Twelve studies included participants who could walk with or without assistance before treatment and ten studies selected participants who could follow simple verbal instructions [22,26,27,28,29,30,31,34,35,37,38,39,40].

### 3.5. Interventions Administered 

Six studies described the effects of PNF intervention alone, compared to either non-intervention [40] or other treatment strategies [22,25,32,33,38], such as treadmill training [22,38], neurodevelopmental treatment (NDT) [32], general exercise [25] and weight-bearing exercise [33]. In these studies, the duration of PNF intervention was from 20 to 30 min per session, with the rehabilitation program including 12 to 36 sessions in total.

There were 13 studies investigating the effects of PNF combined with non-PNF strategies in comparison to electrical stimulation [30,35], conventional physical therapy [24,31,34,39], treadmill training [27], NDT [29], constraint-induced movement therapy [23], eye movement programs [28,36] or taping administered alone [26,37]. The duration of these PNF-based combination therapeutic approaches ranged from 30 to 105 min per session, with the rehabilitation program including a range of sessions from 1 to 40 sessions. One study reported superior immediate effects of PNF combined with kinesio taping on gait function compared with using PNF or taping alone [37]. All the characteristics and reviews of the 19 studies are described in Table 3.

Diverse PNF approaches were employed in the included studies, among which six studies used the PNF pelvic pattern for gait training [23,25,35,38,39,40] and three studies used PNF sprinter and skater patterns for balance and gait velocity training [29,33,34]. Significant improvements in the balance and gait were reported in one study using NDT combined with PNF underwater (involved the sprinter and skate patterns) at a temperature of 32–34 °C and depth of 100 cm when compared to using NDT alone [29].

### 3.6. Outcome Measures

Outcome measures related to motor function, including balance and gait, were used in all the included studies. Identical measurements of BBS, TUG, FRT and 10WMT on balance and gait function were performed in twelve studies [22,23,24,25,26,27,28,29,30,31,33,34] on which meta-analysis was conducted for this review study (Table 3).

#### 3.6.1. Balance

Outcome measures, namely, the frailty and injuries cooperative studies of intervention techniques (FICSIT-4, tests of static balance), four square step test [33], measurement of center of pressure (COP) and velocity moment from force platform [28,32,36]; BBS [22,24,28,29,30,34]; FRT [28,29,31,34] and TUG [26,27,29,30,33,34] were adopted by the included studies to assess balance function. The findings in these studies revealed significant differences in the balance function before and after PNF intervention [22,24,26,27,28,29,30,31,32,33,34,36] and between patients receiving PNF and controls [22,24,26,27,28,29,30,34].

##### Berg Balance Scale

**Figure 3:**
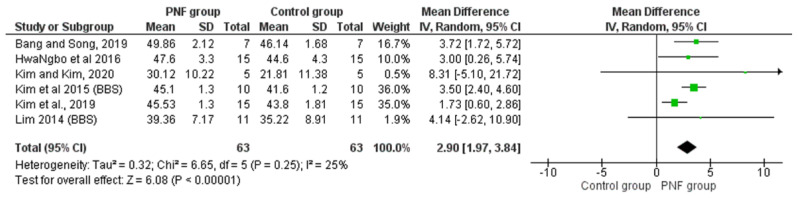
Forest plot of effect of PNF intervention on BBS scores. Abbreviations: IV: inverse variance; CI: confidence interval; SD: standard deviation. Refs. [22,24,28,29,30,34].

The BBS, which has proven reliability and validity, was used to examine the ability of patients to balance before and after the treatment. The BBS consists of 14 items, each of which has a minimum score of 0 and a maximum score of 4; the maximum score is 56. A higher score of BBS indicates good steady state and proactive balance [41]. There were six studies showing significant improvements in BBS scores in patients with chronic stroke receiving PNF-based intervention, compared to those undergoing non-PNF interventions [22,24,28,29,30,34]. Meta-analysis results show significant differences in BBS for participants in the PNF group (*n* = 63) compared to the control group (*n* = 63) (MD = 2.90, 95% CI: 1.97~3.84, *p* < 0.001) with low statistical heterogeneity (*p* = 0.25, I^2^ = 25%, Figure 3).

##### Functional Reach Test

**Figure 4:**
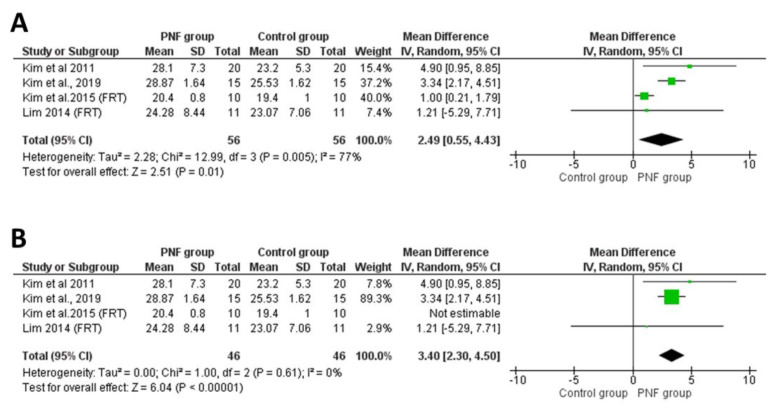
(**A**) Forest plot of effect of PNF intervention on FRT. Refs [28,29,31,34]; (**B**) sensitivity analysis of effect of PNF intervention on FRT. Abbreviations: IV: inverse variance; CI: confidence interval; SD: standard deviation. Refs. [29,30,31,34].

The FRT measures the distance (in centimeters) between the start and end positions while standing independently, raising an arm 90° from the torso and reaching out without losing balance (i.e., taking a step). A far distance of the FRT indicates good proactive balance [42]. Of the 19 studies included, 4 showed significant improvements in the maximal horizontal distances for FRT in patients with chronic stroke after PNF-based intervention compared to those before PNF treatment [31] and in controls after non-PNF interventions [28,29,34]. The meta-analyses showed significant changes in FRT performance between participants in the PNF group (*n* = 56) and controls (*n* = 56) (MD = 2.49 cm, 95% CI: 0.55–4.43, *p* = 0.01) with higher heterogeneity (*p* = 0.005, I^2^ = 77%, Figure 4A). The observed heterogeneity was attributed to the magnitude of the study of Kim et al. [29]. After excluding the study of Kim et al. [29], the overall pooled effect was enhanced (MD = 3.40 cm, 95% CI: 2.30–4.50, *p* < 0.05) with low heterogeneity (*p* = 0.61, I^2^ = 0%, Figure 4B).

##### Timed up and Go Test

**Figure 5:**
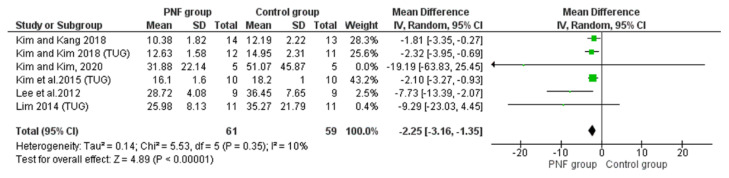
Forest plot of effect of PNF intervention on TUG test. Abbreviations: IV: inverse variance; CI: confidence interval; SD: standard deviation. Refs [26,28,29,33,34].

The TUG test is a performance-based measure of balance and functional mobility. The time taken to sit on an armchair, stand up at the starting signal, walk 3 m and return to the sitting position is measured. The scores of ten seconds or less indicate normal mobility and balance, 11–20 s is within the normal limits for frail, elderly and disabled patients [42]. Of the 19 included studies, 6 studies showed significant differences in TUG in patients with chronic stroke after PNF-based intervention compared to those before treatment [33] and controls with non-PNF interventions [26,27,29,30,34]. Meta-analysis results revealed significant differences between participants in the PNF group (*n* = 61) and controls (*n* = 59) (MD = −2.25 s, 95% CI: −3.16~−1.35, *p* < 0.001) with low heterogeneity (*p* = 0.35, I^2^ = 10%, Figure 5).

#### 3.6.2. Gait

Outcome measures, namely, 10MWT [23,25,26,27,29,40], TUG [29,34], 6-minute walking test [26,27], walking distance per minute [35], kinematic gait parameters [22,30,37,38,39], functional ambulation performance [39], Wisconsin gait scale [40], dynamic gait index and Figure 8 walking test [25] were adopted by the included studies to assess gait function. The findings in these studies reveal significant differences in gait function before and after PNF intervention [22,23,25,26,27,28,29,30,34,35,37,38,39,40] and between patients receiving PNF and controls [22,23,25,26,27,29,30,34,39].

##### 10-Meter Walking Test

**Figure 6:**
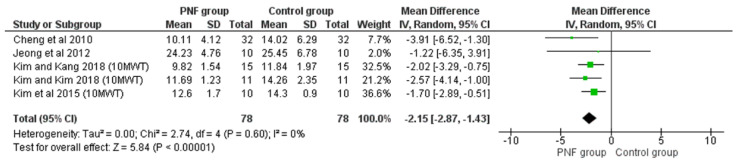
Forest plot of effect of PNF intervention on 10MWT. Abbreviations: IV: inverse variance; CI: confidence interval; SD: standard deviation. Refs. [23,25,26,27,29].

The 10MWT is a performance measure used to assess gait velocity in meters per second over a 10 m distance. It can be employed to determine functional mobility and gait. Good walking-speed performance required for the subject to walk 10 m on a course indicates good functional mobility in individuals with chronic stroke, also as a practical and informative functional sixth “vital sign” for all patients [43]. Of the 19 included studies, 5 showed significant differences in 10MWT performance before and after PNF intervention and between patients with chronic stroke treated with PNF and non-PNF interventions [23,25,26,27,29]. Meta-analysis results also revealed significant differences in 10MWT performance between patients with chronic stroke in the PNF group (*n* = 78) and controls (*n* = 78) (MD = −2.15 s, 95% CI: −2.87~−1.43, *p* < 0.001) with low heterogeneity (*p* = 0.08, I^2^ = 0%, Figure 6).

## 4. Discussion

The current study exclusively focused on the chronic stroke population. To our knowledge, this study is the first systematic review and meta-analysis examining the effects of PNF-based physical therapy on the improvement of balance and gait function in patients with chronic stroke. A previous systematic review and meta-analysis concerning four studies using trunk PNF patterns demonstrated positive effects of PNF on trunk control and balance in both the acute and subacute stages of stroke [44]. Another systematic review with five included studies suggested that PNF improved gait parameters in patients with stroke [11]. The current meta-analysis provides evidence supporting the beneficial effects of the PNF-based physical therapy approach on the improvement of balance and gait velocities with many specific PNF patterns and techniques by assessing 10MWT, BBS, FRT and TUG in patients with chronic stroke. While our findings on balance and gait functions are comparable with the previous results from pooled patients with stroke, mainly at the acute and subacute stages [11,44], the current review demonstrated the positive effects of PNF intervention in strengthening the impaired balance and gait in patients with stroke, specifically at the chronic stage.

This present meta-analysis with a statistical evidence for BBS, FRT and TUG measurements shows that the potential PNF patterns and techniques adopted in the included studies are appropriate for improving static and dynamic balance abilities during postural changes and mobility in patients with stroke, specifically at the chronic stage. Among those studies using BBS, FRT and TUG measurements, diversified PNF patterns were used, including PNF sprinter and skater exercise [25,29,34], neck pattern [22,24,28], scapular and pelvic patterns in a side-lying position [27] and both leg patterns [30]. The positive outcomes obtained suggest that PNF could facilitate core muscle control, which in turn improves balance through coordination movement and enhances balance ability by stimulating a proprioceptive sense of muscles and tendons [45]. The patterns of PNF exercises have a spiral, diagonal direction, which further emphasizes the functional training on trunk stability aiming to enhance balance in a lateral direction [46,47]. The lateral balance of trunk control, which was more affected by stroke than balance in the anteroposterior direction, seems to be a primary target for rehabilitation [48]. BBS and TUG can provide the clinical validity of balance capacity measures, including the performance of lateral, static and dynamic balance control [48,49,50]. In line with the previous results, BBS, FRT and TUG in patients with chronic stroke were found to improve after PNF. Moreover, the beneficial effects of PNF therapy on gait function in patients with chronic stroke were also observed, especially in a walking speed of 10MWT as revealed in the meta-analysis results [23,25,26,27,29,40]. Taken together, the findings suggest that PNF intervention increases lateral, static and dynamic balance to promote functional balance and the mobility of patients with stroke at the chronic stage. The previous studies have shown that balance may be a predictor of gait performance in patients with chronic hemiparetic stroke [51,52], implying a strong correlation between balance and gait parameters. There were four studies in this review presenting the significant effect of PNF on the improvement in balance and gait speed for patients with chronic stroke [26,27,29,34].

Previous studies have shown that the pelvic pattern of PNF helps to improve control of the pelvis, which is crucial for maintaining trunk control, gait and balance through the stimulation of muscle and joint proprioception [53]. Specific core-stability training for patients with stroke would improve not only trunk function, but also balance and mobility. Moreover, it would lead to greater improvement compared to a conventional comprehensive rehabilitation program [54]. Among those studies on gait function, the pelvic pattern of PNF was commonly used in gait training programs [23,25,35,38,39,40], which aim to increase core stability for promoting ambulation in patients with stroke. Several included studies also demonstrated that PNF pattern exercise using sprinter and skater also contributed to enhance the balance and gait functions in patients with chronic stroke [29,33,34,37]. Regarding the duration of the PNF treatment program, a 30-min PNF intervention at least for 12 sessions in most of the included studies has been shown to improve balance and gait abilities in patients with chronic stroke [24,25,33,37,39]. In view of these findings, long-term PNF intervention aiming to promote trunk control and lower-limb strength was recommended for increasing balance ability and walking speed in patients with chronic stroke. PNF can still benefit patients by enhancing their balance and gait abilities at more than 6 months after stroke onset. Hence, the inclusion of PNF in a routine treatment regime of chronic stroke individual can be supported.

Consistent with the previous reviews, the emphasis of this work was on balance and gait functions as major deficits of chronic stroke hampering functional recovery in neurorehabilitation [55,56,57]. Comparative studies with alternative PNF treatments were analyzed in this review, which would shed light on the significant differences of PNF interventions on motor impairments by conducting kinematic parameters, subjective reports or objective measures and activity limitations examined during the chronic stages of recovery. These findings need to be further integrated into current practice recommendations.

Evidence for stroke rehabilitation relating to walking ability, postural control, muscle strength and functional recovery is becoming increasingly available in the form of high-quality RCTs that can inform clinical guidelines as well as high-level government strategies with respect to stroke [58]. Individualized, patient-centered, evidence-based physical treatments, with consideration of all the available treatment components, should be selected by using a mix of components from different approaches. PNF is one of the effective physical interventions to decrease muscle spasticity and improve lower-limb function and gait speed in post-stroke survivors, as well as cycling, treadmill exercise, functional electrical stimulation and deep dry needling [8,9,59].

### 4.1. Limitations of Included Studies

Of the 19 included studies, 6 were of good quality while 10 were of fair quality according to the PEDro scale. Insufficient good-quality studies may influence the precision, applicability and confidence of our results and recommendations. Hence, more quality studies should be included in future research. Moreover, the included studies have the following limitations. First, most studies had a small sample size. Second, the RCTs involved experimental groups receiving PNF with different intervention periods, intervention times, environments and more diverse tasks, which had variations in intervention implementation, making it difficult to interpret the isolated effects of PNF in the treatment of this population. Third, biased results arose from ignoring minimal clinically important differences in each assessment in each trial. It may not seem as though the difference meets the threshold for minimal clinically important differences across the assessments; however, there were statistically significant differences between the experimental and control groups. Fourth, follow-up observation reports were lacking. In view of these limitations, the current results should be interpreted with caution and cannot be generalized to all patients with chronic stroke.

### 4.2. Strengths and Weaknesses of This Review

The current study is an updated systematic review and meta-analysis on the literature published from 1960 to 2021 addressing PNF for people with stroke at a chronic stage. The included studies were not restricted to RCTs published in English, thus offering a more comprehensive understanding on the effect of PNF intervention in the published literature related to balance and gait in patients with chronic stroke.

However, this review has significant limitations. Out of 19 analyzed RCTs, 15 were conducted in Korea and 4 studies in the United States, China, Brazil and Poland, respectively, which may limit generalizability. The included studies covered only RCTs; not including the published clinical trials may be a significant miss and underestimates the effects of PNF on patients with chronic stroke. The small number of RCTs reflects the difficulties in conducting such investigations on patients with stroke at the chronic stage, which may be related to the PNF technique, the study design itself or patient-related or ethical issues. Publication bias may also be another reason for the small sample. Second, different methodologies for evaluating balance and gait functions were used, making it impossible to conduct a meta-analysis of all the selected studies. This reflects the variation in assessments of balance and gait functions affected by PNF interventions in chronic stroke. We attempted to maintain an acceptable level of rigor and quality to recommendations through a similar process of clinical and scientific inquiry to examine the current literature.

### 4.3. Implications for Clinical Practice and Research

At more than 6 months after stroke onset, PNF can still benefit patients by enhancing their balance and gait abilities. In particular, PNF focusing on trunk control in patients with stroke can improve their balance function and ability to walk. Using PNF pelvic, neck, sprinter and skater patterns for trunk control in various positions with isotonic, dynamic reversal, stretching, reversal stabilization or resisted techniques can provide strategy implementation for clinical practice on balance and gait functions at the chronic stage for patients with stroke. It is worth noting that the effect of PNF on patients with chronic stroke is not based on short-term effects, but requires long-term continuous treatment to obtain more persistent benefits. The findings of this review indicate that there were statistically significant improvements to the balance and gait speed for patients with chronic stroke. However, a statistically significant difference does not necessarily mean that it ensures a clinically meaningful difference as well. Further high-quality research with the best available evidence is required for concluding a consensus of clinical intervention and research on PNF.

## 5. Conclusions

The most significant recovery of movement is generally considered to occur within the first six months following a stroke, with spontaneous recovery slowing down after that time. That does not mean that patients with chronic stroke should be prevented from addressing intensive therapy for motor recovery after six months. The results from this systematic review with meta-analysis suggest that PNF-based physical therapy has statistical effects on the improvement of balance and gait speed in individuals at least 6 months after a stroke. Although positive statistical effects were found in this study, more advancing rehabilitation studies with the sciences of neuroplasticity are needed for concluding a consensus of the clinical and research significances of data regarding the relative efficacy of PNF, including the technique components, dosage parameters and practice conditions in future meta-analyses.

## Figures and Tables

**Table 1:** Search strategies and keywords list.

Main Term	Keyword
#1 Population	“Stroke” OR “Cerebrovascular accident” OR “Cerebrovascular disease” OR “Cerebrovascular disorders” OR “CVA” OR “Hemiplegic” OR “Hemiplegia”
#2 Intervention	“Proprioceptive Neuromuscular Facilitation” OR “PNF” OR “Proprioceptive” OR “Neuromuscular Facilitation”
#3 Outcomes	“Gait” OR “Walking” OR “Ambulation” OR “Lower extremity” OR “Endurance” OR “Balance” OR “Mobility” OR “Posture” OR “Postural control”
#4 Final Search	#1 AND #2 AND #3

**Table 2:** Methodological quality of included studies on PEDro scale.

	Scale Item											
Study/Author	Eligibility ^†^	Randomization	Allocation Concealment	Comparison at the Baseline	Blinded Participants	Blinded Therapists	Blinded Evaluators	Adequate Follow-Up	Intention-to-Treat Analysis	Comparisons between Groups	Measures of Precision and Variability	Total Score
Bang and Song, 2019 [22]		✓		✓				✓		✓	✓	5
Cheng et al., 2010 [23]	✓	✓		✓				✓		✓	✓	5
Hwangbo and Kim 2016 [24]	✓	✓		✓				✓		✓	✓	5
Joeng et al., 2012 [25]		✓		✓				✓		✓	✓	5
Kim and Kim 2018 [26]	✓	✓		✓		✓		✓		✓	✓	6
Kim and Kang 2018 [27]		✓		✓				✓		✓	✓	5
Kim and Kim, 2020 [28]		✓		✓				✓		✓	✓	5
Kim et al., 2015 [29]	✓	✓		✓				✓		✓	✓	5
Kim et al., 2020 [30]		✓		✓				✓		✓	✓	5
Kim et al., 2011 [31]		✓		✓				✓		✓	✓	5
Krukowska et al., 2016 [32]	✓	✓		✓				✓		✓	✓	5
Lee et al., 2012 [33]	✓	✓		✓				✓		✓	✓	5
Lim 2014 [34]	✓	✓		✓			✓	✓		✓	✓	6
Moon et al., 2010 [35]	✓	✓		✓				✓	✓	✓	✓	6
Park et al., 2016 [36]	✓	✓		✓				✓		✓	✓	5
Park 2017 [37]	✓	✓		✓				✓		✓	✓	5
Ribeiro et al., 2013 [38]	✓	✓		✓			✓	✓	✓	✓	✓	7
Seo et al., 2012 [39]	✓	✓	✓	✓				✓		✓	✓	6
Stephenson et al., 2014 [40]	✓	✓		✓			✓	✓		✓	✓	6

✓: “yes”; †: does not contribute to total score.

**Table 3:** Characteristics and outcome reviews of included studies.

RCT Study Author, Year	Number of Participants (Mean Age in Years)	Grouping and Intervention (Time in Minutes)	Total Sessions (Times/Week)	Outcome Measures	Significant Improvement *
Bang and Song 2019 [22]	14 EG: 7 (58.86 ± 6.49) CG: 7 (57.71 ± 6.70)	EG: PNF (20) CG: treadmill (20) *^#^ PNF* *neck pattern*	20 (5 for 4 weeks)	Balance: BBS Gait parameters	BBS and in terms of gait speed, cadence, step length, and double-limb
Cheng et al., 2010 [23]	64 EG: 32 (52.3 ± 9.5) CG: 32 (51.7 ± 10.3)	EG: PNF + CIMT (45) CG: CIMT (30-60) *^#^* *PNF trunk and limbs, gait patterns with resistance, stretch, rhythmic stability, dynamic reversal*	40 (5 for 8 weeks)	Upper limb function and fine motor evaluation: STEF Gait velocity: 10MWT ADL: MBI	STEF, 10MWT and MBI: EG.
Kim et al., 2011 [31]	40 EG: 20 (51.4 ± 5.7) CG: 20 (53.5 ± 7.1)	EG: PNF (10) + general therapeutic exercise (20) CG: general therapeutic exercise (30)	30 (5 for 6 weeks)	Trunk stability: FRT Muscle activity: EMG	FRT, activities of soleus and quadriceps: EG
		^#^ *PNF* *stabilizing reversal and Rhythmic stabilization (sitting* *, standing)*			
Hwangbo and Kim, 2016 [24]	30 EG: 15 (59.4 ± 9.1) CG: 15 (55.9 ± 9.8)	EG: PNF (30) + traditional rehabilitation (30) CG: traditional rehabilitation (60)	30 (5 for 6 weeks)	Trunk control: TIS Balance: BBS	BBS, dynamic sitting, coordination and TIS: EG
		*^#^ PNF neck pattern (sitting)*			
Kim and Kang 2018 [26]	27 EG: 14 (51.4 ± 2.6) CG: 13 (51.5 ± 2.9)	EG: treadmill with PNF lower-leg taping (30) CG: treadmill with placebo lower-leg taping (30) *^#^ PNF flexion–adduction–external rotation pattern*	30 (5 for 6 weeks)	Balance: TUG Walking ability: 10MWT and 6MWT	TUG, 10MWT and 6MWT: EG
Kim and Kim 2018 [27]	23 EG: 12 (60.8 ± 3.1) CG: 11 (60.6 ± 3.4)	EG: PNF (15) + treadmill (15) CG: treadmill training (30) *^#^ PNF scapular and pelvic patterns with hold-relax, contract-relax, and dynamic reversal (sidelying)*	30 (5 for 6 weeks)	Balance: TUG Walking ability: 10MWT and 6MWT	TUG, 10MWT and 6MWT: EG
Kim and Kim 2020 [28]	10 EG: 5 (70.61 ± 13.08) CG: 5 (71.00 ± 6.02)	EG: PNF (30) + functional electrical stimulation (30) CG: general physical therapy (30) + functional electrical stimulation (30) *^#^PNF bilateral lower extremity asymmetric flexion/extension patterns*	20 (5 for 4 weeks)	Balance: BBS, TUG Gait parameters	Balance: BBS, TUG Gait Velocity
Kim et al., 2015 [29]	20 EG: 10 (65.9 ± 6.2) CG: 10 (64.1 ± 3.6)	EG: PNF underwater (8) + NDT (30) CG: NDT (30)	30 (5 for 6 weeks)	Balance: BBS and FRT Gait velocity: 10MWT Gait function: TUG	BBS, FRT, 10MWT and TUG: EG
		*^#^ PNF sprinter and skate patterns (standing, underwater)*			
Kim et al., 2020 [30]	30 EG: 15 (56.8 ± 3.44) CG: 15 (57.53±3.59)	EG: eye movement (15) + PNF (15) + conservative treatment (30) CG: conservative treatment (60)	24 (3 for 8 weeks)	Trunk control: TIS Balance: COP, LOS, BBS, FRT	TIS, COP, LOS, BBS, FRT
		*^#^PNF neck pattern movement training*			
Krukowska et al., 2016 [32]	72 EG: 34 (52.7 ± 7.5) CG: 39 (52.7 ± 6.3)	EG: PNF ^†^ CG: Bobath-NDT ^†^ *^#^PNF diagonal and spiral patterns*	36 (6 for 6 weeks)	Balance: force platform (COP of field support and total path length)	Movement of COP: CG
Lee et al., 2012 [33]	27 EG1: 9 (49.1 ± 9.0) EG2: 10 (51.7 ± 17.4) CG1: 9 (44.8 ± 8.6)	EG1: PNF (30) EG2: functional weight bearing exercise (30) CG: general exercise (30) *^#^* *PNF sprinter pattern (side lying, half standing, modified plantigrade posture)*	12 (3 for 4 weeks)	Weight bearing and static balance capability: FICSIT-4, force platform Dynamic balance: FSST and TUG	FICSIT-4, GBS: EG1, EG2 FSST, TUG: EG1, EG2
Lim 2014 [34]	22 EG: 11 (55.5 ± 5.4) CG: 11 (56.4 ± 5.7)	EG: PNF (15) + conventional physical therapy (35) CG: conventional physical therapy (50) ^#^ *PNF pattern sprinter and skater (sitting and half standing)*	20 (5 for 4 weeks)	Balance: FRT and BBS Gait function: TUG	FRT, BBS and TUG: EG
Moon et al., 2010 [35]	15 aPNFG: 5 (49.8 ± 2.9) cPNFG: 5 (53.4 ± 2.5) CG: 5 (52.4 ± 7.1)	aPNFG: PNF (30) cPNFG: PNF (15) + ES (15) CG: ES (30) *^#^PNF scapula + upper limb- combining-isotonic- dynamic reversal (side lying, sitting)*	30 (5 for 6 weeks)	Upper limb functions: MFT Gait velocity: walking distance/minutes Weight bearing: force platform	Upper limb function: aPNFG
Park et al., 2016 [36]	20 EG: 10 (61.1 ± 8.2) CG: 10 (60.2 ± 7.9)	EG: PNF (30 times) + EMP (20 times) ^†^ CG: EMP (20 times) ^†^ ^*#*^ *PNF neck pattern with contract-relax (sitting)*	30 (5 for 6 weeks)	Balance: force platform (Static and dynamic balance: sway length and area with eye open/closed; Dynamic balance: limit of stability with forward/backward and left/right) Head alignment: GPS	Static balance with eye closed: EG Head alignment: EG
Park 2017 [37]	20 aPNFG: 7 (57.3 ± 9.4) cPNFG: 7 (51.7 ± 6.5) CG: 6 (64.8 ± 15.2)	aPNFG: PNF (30) cPNFG: PNF (30) + kinesio taping CG: kinesio taping	1	Gait parameters	Cadence, speed, and stride length: cPNFG
		*^#^ PNF sprinter and skater patterns (sitting, standing)*			
Ribeiro et al., 2013 [38]	20 EG: 9 (58.3 ± 8.9) CG: 11(56.5 ± 8.3)	EG: PNF (30) CG: treadmill with partial body weight support (30)	12 (4 for 3 weeks)	Motor recovery and basic mobility: STREAM ADL: Motor FIM Gait parameters	Ankle dorsiflexion during swing phase: EG
		*^#^ PNF scapular and pelvic patterns (sidelying, sitting and standing with stretching and maximum resistance)*			
Seo et al., 2012 [39]	40 EG: 20 (64.1 ± 3.2) CG: 20 (65.8 ± 6.0)	EG: PNF-based walking exercise (30) + general physical therapy (30) CG: general physical therapy (30) + walking exercise (30)	20 (5 for 4 weeks)	Gait function: temporal, spatial parameters and FAP	All parameters of gait performance and FAP: EG
		*^#^ PNF gait training*			
Stephenson et al., 2014 [40]	18 EG: 6 (63.3 ± 12.4) CG1: 6 (55.0 ± 9.4) CG2: 6 (63.8 ± 12.2)	EG: PNF gait training (30) CG1: treadmill with body weight support (20) CG2: no interventions *^#^ PNF pelvic and lower extremity patterns and gait training*	12 (3 for 4 weeks)	Gait velocity and cadence: 10MWT Gait disability: WGS	Gait velocity and cadence: EG > CG1 WGS total score: EG

The 10-m walking test (10MWT); 6-minute walk test (6MWT); activity of daily living (ADL); Berg balance scale (BBS); center of pressure (COP); constraint-induced movement therapy (CIMT); Dynamic gait index (DGI); eye movements program (EMP); Figure 8 walking test (F8W); four Square step test (FSST); frailty and injuries cooperative studies of intervention techniques (FICSIT-4); functional ambulation performance (FAP); functional independence measure (FIM); functional reach test (FRT); global postural system (GPS); good balance system (GBS); modified Barthel index (MBI); PNF-alone group (aPNFG); PNF-combined group (cPNFG); simple test for evaluating hand function (STEF); the stroke rehabilitation assessment of movement (STREAM); timed up-and-go test (TUG); trunk impairment scale (TIS). *^#^* PNF techniques in italic * Indicates significant differences compared to other groups. ^†^ Indicates no time data provided by authors.

## Data Availability

Not applicable.
